# Endurance Training-Induced Increase in Circulating Irisin Levels Is Associated with Reduction of Abdominal Visceral Fat in Middle-Aged and Older Adults

**DOI:** 10.1371/journal.pone.0120354

**Published:** 2015-03-20

**Authors:** Eri Miyamoto-Mikami, Koji Sato, Toshiyuki Kurihara, Natsuki Hasegawa, Shumpei Fujie, Satoshi Fujita, Kiyoshi Sanada, Takafumi Hamaoka, Izumi Tabata, Motoyuki Iemitsu

**Affiliations:** 1 Faculty of Sport and Health Science, Ritsumeikan University, Shiga, Japan; 2 Japan Society for the Promotion of Science, Tokyo, Japan; Medical University Innsbruck, AUSTRIA

## Abstract

To elucidate the effects of endurance training on circulating irisin levels in young and middle-aged/older adults, and to determine the association between endurance training-induced alteration of irisin and reduction in body fat. Twenty-five healthy young (age 21 ± 1 years; 16 men, 9 women) and 28 healthy middle-aged/older adults (age 67 ± 8 years; 12 men, 16 women) participated in the study. Each age cohort was divided into two groups: the endurance-training group (14 young, 14 middle-aged/older) and the control group. Subjects in the training groups completed an 8-week endurance-training program (cycling at 60-70% peak oxygen uptake [$\dot{\text{V}}$O_2peak_] for 45 min, 3 days/week). Before and after the intervention, we evaluated serum irisin level, $\dot{\text{V}}$O_2peak_, and body composition. The increase in $\dot{\text{V}}$O_2peak_ in the young and middle-aged/older training groups after the intervention period was significantly greater than those in the young and middle-aged/older control groups (*P* < 0.05). Serum irisin level was significantly increased in the middle-aged/older training group after the intervention period (*P* < 0.01), but not in the young training group. Furthermore, in the middle-aged/older training group, the endurance training-induced reduction in visceral adipose tissue area was negatively correlated with the change in serum irisin level (r = −0.54, *P* < 0.05). These results suggest a possible role for secreted irisin in the exercise-induced alteration of abdominal visceral fat in middle-aged and older adults.

## Introduction

Fat accumulation induces obesity and increases the risk of type 2 diabetes, cardiovascular diseases, hypertension, and dyslipidemia. Regular physical exercise reduces or prevents fat accumulation throughout the body, in particular as visceral fat [1–3], thereby reducing the risk of these lifestyle-related diseases. Recent studies have shown that myokines produced and released by exercise may participate in regulation of fat oxidation [4].

Expression of fibronectin type III domain-containing 5 (FNDC5) protein is elevated upon stimulation of peroxisome proliferator-activated receptor (PPAR)-γ co-activator (PGC)-1α [5]. The *FNDC5* gene encodes a type I membrane protein that is proteolytically cleaved and subsequently secreted into the blood as a cytokine called irisin. Bostrom et al. [5] demonstrated that irisin secreted by skeletal muscle upregulates uncoupling protein 1 (*UCP1*) gene expression via activation of PPAR-α protein in white adipose cells, thereby increasing energy expenditure through thermogenesis. Additionally, in healthy post-menopausal women, circulating irisin levels are positively correlated with 24-h energy expenditure in subjects whose energy expenditure is greater than predicted by the fat free mass-based equation [6]. Therefore, a high level of circulating irisin may be associated with resistance to fat accumulation throughout the body. However, given that FNDC5 expression in muscle [7, 8] and circulating irisin [9] are positively correlated with body mass index (BMI), it is unclear whether irisin plays a role in exercise training-induced reduction of body fat accumulation.

Circulating irisin levels decrease with age [9, 10], whereas circulating irisin and muscle FNDC5 expression increases with endurance training in older adults [5]. Additionally, *FNDC5* mRNA expression in muscle is higher in elderly high-fitness men than in elderly low-fitness men, but does not differ between young high- and low-fitness men [8]. Thus, it is possible that the aging-induced reduction in circulating irisin level can be restored by chronic endurance training, and that this effect might be age-specific.

We hypothesized that endurance training might increase circulating irisin levels in older adults, and that this increase in irisin might contribute to the endurance training-induced reduction in accumulation of fat throughout the body, particularly visceral fat. To test our hypothesis, we measured serum irisin levels and whole-body fat accumulation in healthy young and middle-aged/older adults before and after an exercise intervention trial. In addition, we examined the associations between the effects of training on circulating irisin levels and accumulation of fat, including whole-body fat, visceral fat, and subcutaneous fat in the abdomen and limbs.

## Materials and Methods

### Subjects

Twenty-five healthy young subjects (age 21 ± 1 years; 16 men, 9 women) and 28 healthy middle-aged/older adults (age 67 ± 8 years, 12 men, 16 women) participated in this present study. Each subject was assigned to either the endurance-training group (n = 14 for both age groups) or the control group. The study population consisted of sedentary or moderately active subjects who did not participate in vigorous sports activities. The mean numbers of daily steps per day were 8567.4 ± 3123.5 steps/day and 6832.8 ± 2431.9 steps/day in the young and middle-aged/older adults, respectively. The mean numbers of daily steps in the present study were similar to the reference values included in the report researched by the Ministry of Health, Labor, and Welfare of Japan for National Health and Nutrition Survey (http://www.mhlw.go.jp/bunya/kenkou/kenkou_eiyou_chousa.html). Subjects taking anti-hyperlipidemic, anti-hypertensive, or anti-hyperglycemic medication, or with a history of stroke, diabetes, hypertension, hyperlipidemia, cardiac disease, chronic renal failure, or mental disorder, were excluded from the study. All subjects were given an oral and written briefing of the study, and written informed consent was obtained from each subject. The study was approved by the Ethics Committees of the Ritsumeikan University and was conducted in accordance with the Declaration of Helsinki.

### Study design

Before and after the 8-week endurance training period, body composition, serum irisin concentrations, and peak oxygen uptake (VO_2peak_) were measured. At the beginning and end of the study period, fasting blood samples were drawn following at least 48 h of rest after the last exercise-training session. All subjects were instructed not to eat or drink fluids other than water for at least 12 h prior to blood sampling. Blood samples were immediately centrifuged (1500 x *g*, 15 min, 4°C), and serum samples were stored at −80°C until use. Room temperature was maintained at 22°C throughout the experiment.

### Endurance training intervention

After baseline testing, young and middle-aged/older training groups participated in an endurance-training program consisting of cycling on a leg ergometer (828E Monark cycle ergometer, Stockholm, Sweden) for 55 min, 3 days/week, for 8 weeks. Each exercise session consisted of a 5-min warm-up at 40% peak oxygen uptake (VO_2peak_), followed by 45 min of cycling at a resistance that elicited 60–70% VO_2peak_, and ended with a 5-min cool-down at 40% VO_2peak_. Exercise training was conducted in the group at 9:00–11:00 AM after breakfast. Exercise compliance was carefully monitored by direct supervision. The young and middle-aged/older control subjects were instructed to maintain the activities of daily living. Subjects in both groups were encouraged to maintain their usual levels of food intake during the experimental period.

### VO_2peak_

VO_2peak_ was measured using an incremental cycle exercise test on a cycle ergometer (828E Monark cycle ergometer). Incremental cycle exercise began at a work rate of 60 W (30–90 W) for men and 30 W (0–60 W) for women, and power output was increased by 15 W·min^−1^ until the subjects could not maintain a fixed pedaling frequency of 60 rpm. The subjects were encouraged during the ergometer test to exercise at maximum intensity. Heart rate and rating of perceived exertion (RPE) were monitored minute by minute during the exercise. RPE was obtained using the modified Borg scale. VO_2_ was monitored during breath-by-breath assessment using a respiratory gas analyzer (Aeromonitor AE-310SRD, MINATO, Osaka, Japan). The highest 30-sec averaged value of VO_2_ values during the exercise test was designated as VO_2peak_ if three out of four of the following criteria were met: (I) plateau in VO_2_ with an increase in external work, (II) maximal respiratory exchange ratio ≥ 1.1, (III) maximal heart rate ≥ 90% of the age predicted maximum (208 − 0.7 × age) [11], and (IV) RPE ≥ 18.

### Body composition

Whole-body fat mass, arm and leg fat mass, percent body fat (% fat), and whole-body fat-free mass were assessed by dual-energy x-ray absorptiometry (DXA; Lunar iDXA, GE Healthcare UK Ltd, Buckinghamshire, UK). Cross-sectional abdominal visceral and subcutaneous adipose tissue areas were assessed by magnetic resonance imaging (MRI; Signa HDxt, 1.5T, GE Healthcare UK Ltd). Subjects lay supine and relaxed, and abdominal transverse image acquisition was synchronized with their respiration. Images were taken using an 8-channel body array coil with the following parameters: echo time/repetition time = 7 ms/respiration; matrix = 384 × 384; field of view = 420 × 420 mm; slice thickness = 10 mm; gap = 0 mm; number of excitations = 2. The intervertebral level between the third and fourth lumbar (L3-L4) spaces was selected for the analyses of visceral and subcutaneous adipose tissue areas, and the cross-sectional areas of the visceral and subcutaneous adipose tissue were determined using image analysis software (Slice-o-matic version 4.3 for Windows, Tomovision, Montreal, Canada). Visceral adipose tissue was defined as the adipose tissue in the region enclosed by the inner aspect of the abdominal wall and the anterior margin of the vertebral body, including the intra-abdominal cavity [12].

### Circulating irisin concentration

Serum irisin concentrations were determined using a commercially available Irisin Enzyme Immunoassay Kit (EK-067-16, Phoenix Pharmaceuticals, Burlingame, CA, USA), according to the manufacturer's protocol. All samples were assayed in duplicate. Optical density at 450 nm was measured using a microplate reader (xMark microplate spectrophotometer, Bio-Rad Laboratories, Hercules, CA, USA). Serum irisin concentrations in pre and post samples of training and control groups were simultaneously determined after completion of the intervention period. In this study, interobserver and intraobserver variabilities of measurements were examined in two observers and 2 kits of ELISA. Interobserver and intraobserver variabilities of measurements were 6.2% and 5.9% for irisin concentration, respectively.

### Statistical Analyses

Values are expressed as means ± SD. Comparisons between the two groups of each parameter at baseline, as well as comparisons of the amount of change and percent change, were conducted by unpaired Student's t-tests. Comparisons of each parameter before and after training intervention were conducted by paired Student's t-test. The influences of groups (training and control) and the two time points (pre and post) on serum irisin concentration were evaluated by two-way repeated-measure ANOVA separately in young and middle-aged/older adults, followed by a Fisher's post hoc test that was applied when a significant interaction was identified. Relationships between the amount of change in serum irisin and each body composition parameter were determined using the Pearson correlation coefficient. A *P* value < 0.05 was considered statistically significant. All analyses were performed using StatView version 5.0 (SAS Institute Inc., Tokyo, Japan) and SPSS version 20.0 (SPSS Inc., Chicago, IL). We calculated required sample size by using Power and Sample Size Calculations Version 3.0, and then the required sample size to detect the training response of irisin concentration in this study was 10 (α = 0.05 and power = 0.8).

## Results

### Comparison of baselines in the training and control groups

At baseline, we observed no statistically significant differences in any parameter between the control and training groups in either age cohort (*P* > 0.05, Tables 1 and 2). In the young training group, body weight, % fat, whole-body fat mass, and abdominal subcutaneous adipose tissue area were significantly decreased, and VO_2peak_ was significantly increased after endurance training (*P* < 0.05, Table 1). Decreases in body weight, % fat, whole-body fat mass, and abdominal subcutaneous adipose tissue area in the young training group were significantly greater than those in the young control group (*P* < 0.05, Table 1). In addition, the increase in VO_2peak_ in the young training group after endurance training was significantly greater than that in the young control group (*P* < 0.05, Table 1).

**Table 1:** Subject characteristics in young control and training groups.

	Young control (n = 11)			Young training (n = 14)		
	Pre	Post	Δ	Pre	Post	Δ
Sex (men/women)	6/5	-		10/4	-	
Age (years)	21 ± 1	-		21 ± 1	-	
Height (cm)	168.3 ± 10.8	-		170.1 ± 11.0	-	
Body weight (kg)	62.1 ± 9.0	62.9 ± 9.6	0.8 ± 1.4	63.8 ± 10.8	63.2 ± 11.1 *	−0.6 ± 1.0 †
BMI (kg/m^2^)	23.1 ± 3.4	23.4 ± 3.6	0.2 ± 0.7	21.9 ± 1.7	21.7 ± 2.0	−0.2 ± 0.4 †
% fat (%)	22.6 ± 8.9	23.3 ± 8.6	0.7 ± 1.4	17.9 ± 6.9	17.2 ± 6.5 *	−0.7 ± 1.1 †
Whole-body fat mass (kg)	13.1 ± 4.6	13.7 ± 4.6	0.5 ± 1.0	10.7 ± 4.2	10.2 ± 3.8 *	−0.6 ± 0.8 †
Whole-body fat-free mass (kg)	46.4 ± 10.2	46.2 ± 10.2	−0.1 ± 0.9	50.3 ± 10.4	50.2 ± 10.4	−0.07 ± 0.7
Arm fat mass (g)	1135.0 ± 516.2	1152.1 ± 517.8	17.1 ± 83.7	863.0 ± 379.6	812.6 ± 362.9	−50.4 ± 95.3
Leg fat mass (g)	4805.0 ± 1875.6	4900.4 ± 1905.4	95.5 ± 255.1	3770.5 ± 1501.0	3683.4 ± 1377.1	−87.1 ± 246.2
Abdominal visceral adipose tissue (cm^2^)	26.0 ± 7.8	26.5 ± 7.4	0.5 ± 5.4	29.3 ± 8.4	26.9 ± 9.0	−2.5 ± 7.2
Abdominal subcutaneous adipose tissue (cm^2^)	95.1 ± 38.6	101.0 ± 43.4	5.9 ± 13.6	86.1 ± 36.5	78.4 ± 32.3 *	−7.7 ± 10.2 †
VO_2peak_ (ml/kg/min)	39.6 ± 6.9	40.3 ± 7.8	0.7 ± 4.5	44.0 ± 7.8	48.2 ± 7.6 *	4.2 ± 2.4 †
Serum irisin concentration (ng/ml)	165.8 ± 21.2	164.5 ± 37.8	−1.2 ± 28.2	155.3 ± 17.3	157.0 ± 17.0	1.8 ± 14.9

BMI: body mass index, VO_2peak_: peak oxygen uptake, Δ: amount of change. Data are expressed as means ± SD.

*P < 0.05: Pre vs. Post

†*P* < 0.05: young control group vs. young training group.

**Table 2:** Subject characteristics in middle-aged/older control and training groups.

	Middle-aged/older control (n = 14)			Middle-aged/older training (n = 14)		
	Pre	Post	Δ	Pre	Post	Δ
Sex (men/women)	6/8	-		6/8	-	
Age (years)	69 ± 6	-		65 ± 8	-	
Height (cm)	160.0 ± 9.0	-		160.1 ± 9.1	-	
Body weight (kg)	56.9 ± 12.6	56.9 ± 12.9	0.0 ± 1.0	60.9 ± 12.0	60.8 ± 12.1	−0.1 ± 0.8
BMI (kg/m^2^)	22.1 ± 3.8	22.1 ± 3.9	0.0 ± 0.4	23.7 ± 3.9	23.6 ± 4.0	−0.1 ± 0.3
% fat (%)	24.7 ± 7.6	24.9 ± 8.3	0.2 ± 2.1	29.8 ± 9.8	28.9 ± 10.2 *	−0.9 ± 1.0
Whole-body fat mass (kg)	13.8 ± 6.7	14.0 ± 7.2	0.2 ± 1.1	18.0 ± 8.2	17.5 ± 8.4 *	−0.6 ± 0.8 †
Whole-body fat-free mass (kg)	40.7 ± 8.1	40.4 ± 8.0	−0.2 ± 0.8	41.0 ± 7.7	41.4 ± 7.7	0.3 ± 0.7
Arm fat mass (g)	1276.9 ± 522.1	1330.5 ± 610.9	53.6 ± 149.4	1612.9 ± 685.9	1558.4 ± 736.0	−54.5 ± 128.0 †
Leg fat mass (g)	3942.7 ± 1677.8	3970.4 ± 1695.1	27.7 ± 412.1	5194.1 ± 2244.5	5166.5 ± 2351.3	−27.6 ± 238.0
Abdominal visceral adipose tissue (cm^2^)	73.1 ± 46.0	77.3 ± 50.2	4.2 ± 16.2	96.7 ± 66.8	81.5 ± 51.4 *	−15.2 ± 22.2 †
Abdominal subcutaneous adipose tissue (cm^2^)	102.7 ± 44.3	100.0 ± 55.4	−2.7 ± 21.8	141.0 ± 66.9	140.1 ± 75.5	−0.9 ± 19.5
VO_2peak_ (ml/kg/min)	24.7 ± 4.4	25.3 ± 5.0	0.6 ± 1.8	24.8 ± 5.1	29.8 ± 6.0 *	5.0 ± 2.7 †
Serum irisin concentration (ng/ml)	142.8 ± 8.7	144.4 ± 9.2	1.6 ± 10.7	140.6 ± 26.7	168.7 ± 21.3 *	28.1 ± 27.8 †

BMI: body mass index, VO_2peak_: peak oxygen uptake, Δ: amount of change. Data are expressed as means ± SD.

*P < 0.05: Pre vs. Post

†*P* < 0.05 middle-aged/older control group vs. middle-aged/older training group.

In the middle-aged/older training group, % fat, whole-body fat mass, and abdominal visceral adipose tissue were significantly decreased, and VO_2peak_ was significantly increased after endurance training (*P* < 0.05, Table 2). Decreases in whole-body fat mass and abdominal visceral adipose tissue area were significantly greater in the middle-aged/older training group than in the middle-aged/older control group (*P* < 0.05, Table 2). In addition, the increase in VO_2peak_ in the middle-aged/older training group after endurance training was significantly greater than that in the middle-aged/older control group (*P* < 0.05, Table 2).

### Comparison of serum irisin concentrations between training and control groups

**Fig 1:**
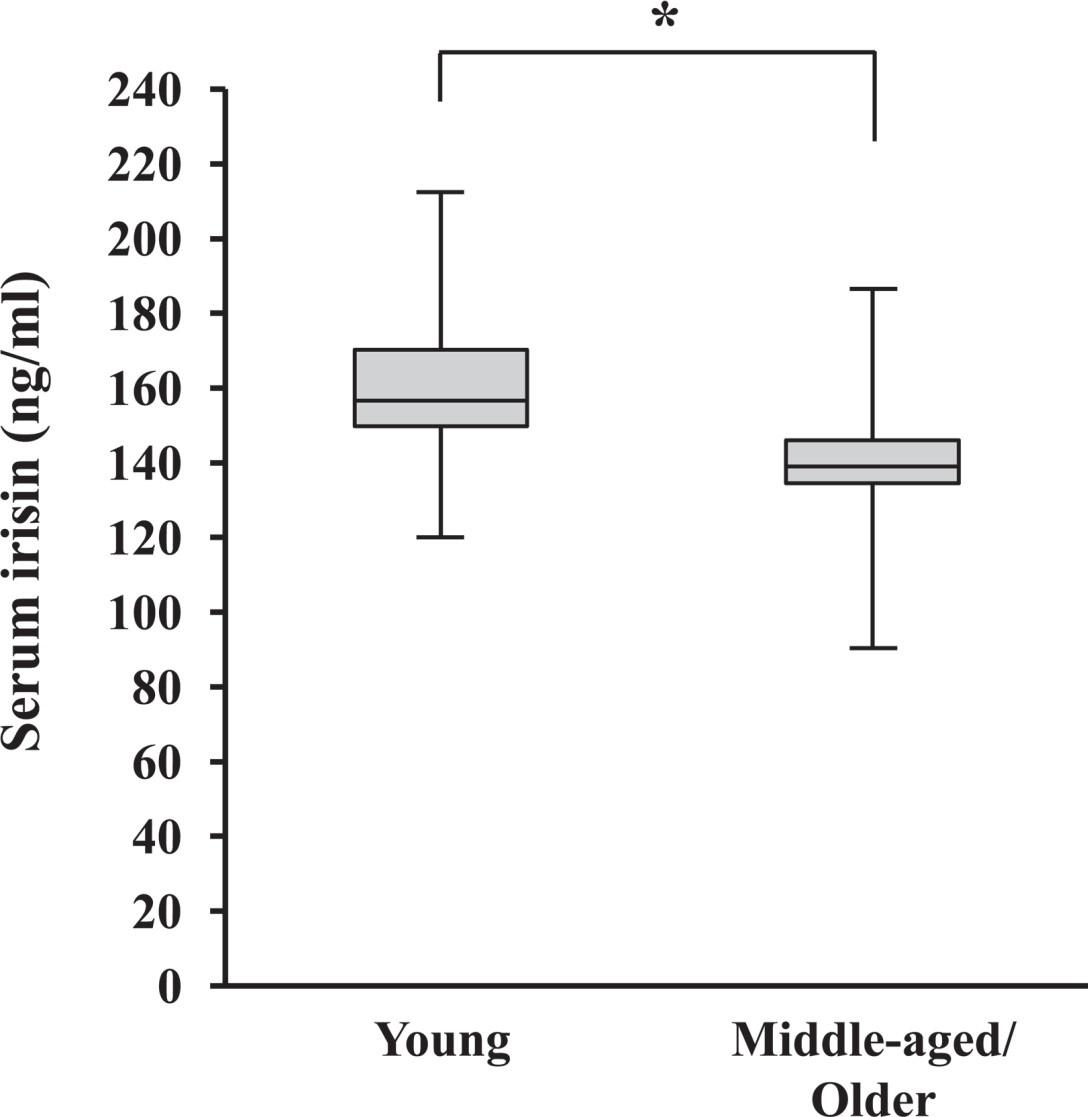
Comparison of serum irisin concentration at baseline between young (n = 25) and middle-aged/older (n = 28) adults. *: *P* < 0.01.

**Fig 2:**
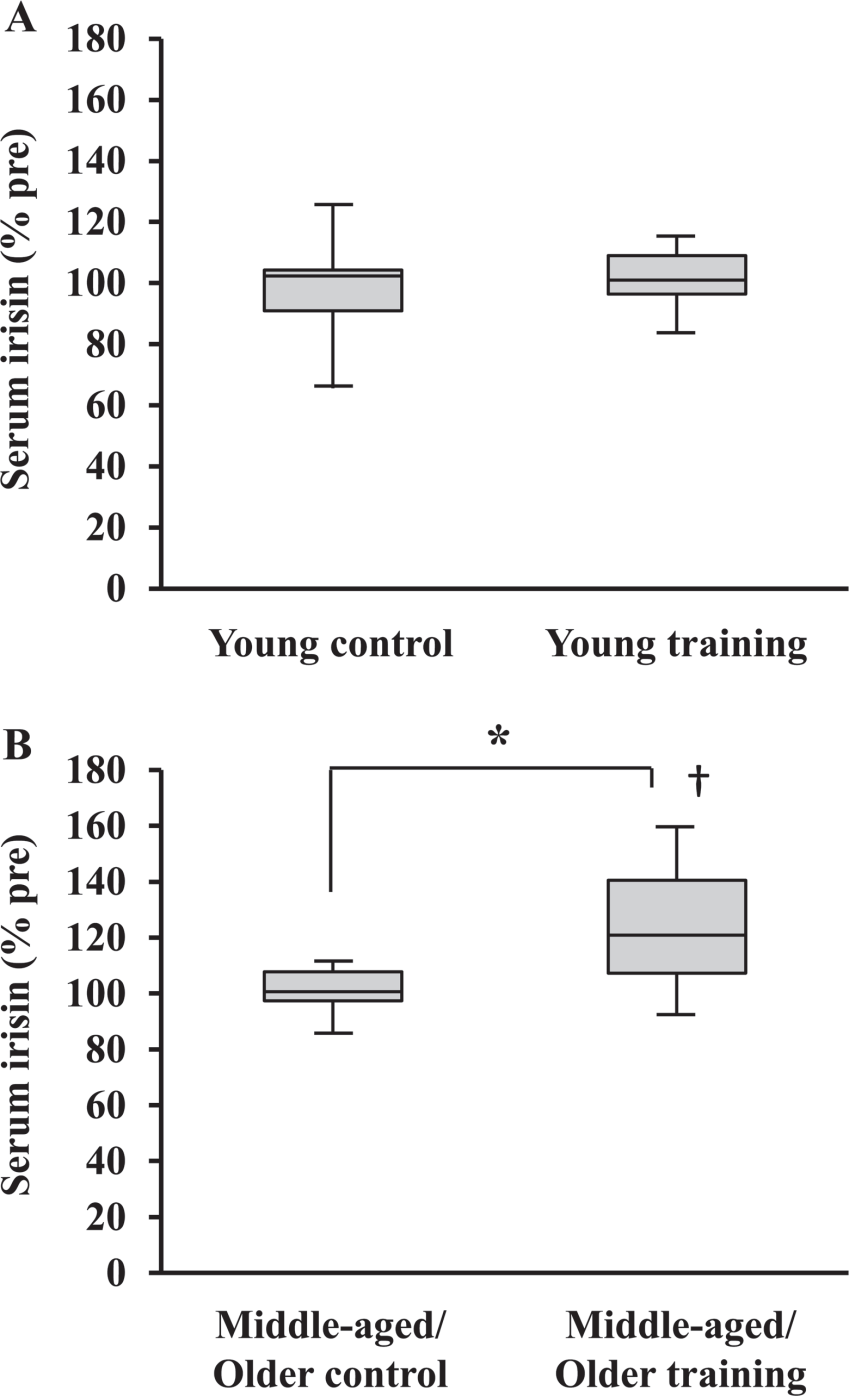
Relative change of serum irisin concentrations in young (A) and middle-aged/older (B) adults before and after the 8-week intervention period. Data are expressed as Post value of irisin concentration normalized with Pre value for each subject.*: *P* < 0.01. †: *P* < 0.05 vs. Pre value in middle-aged/older training group.

Serum irisin concentrations were significantly lower in middle-aged/older subjects than in young subjects (*P* < 0.01, Fig. 1). Before the exercise-training intervention, there were no significant differences in serum irisin concentrations between training and control groups in either age cohort (*P* < 0.05, Tables 1 and 2). However, when the baseline values were normalized to 100, we did observe a significant interaction of groups and time points with relative serum irisin levels in middle-aged/older subjects (*P* < 0.01, Fig. 2-B). After exercise-training intervention in middle-aged/older subjects, serum irisin levels were significantly elevated in the training group (*P* < 0.01, Fig. 2-B and Table 2). Moreover, the percent change in serum irisin concentrations was also significantly greater in the middle-aged/older training group than in the middle-aged/older control group (*P* < 0.01). In young subjects, we observed no significant interactions of groups and time points with relative serum irisin levels (*P* = 0.6218, Fig. 2-A).

### Correlation between serum irisin concentrations and body composition parameters

**Fig 3:**
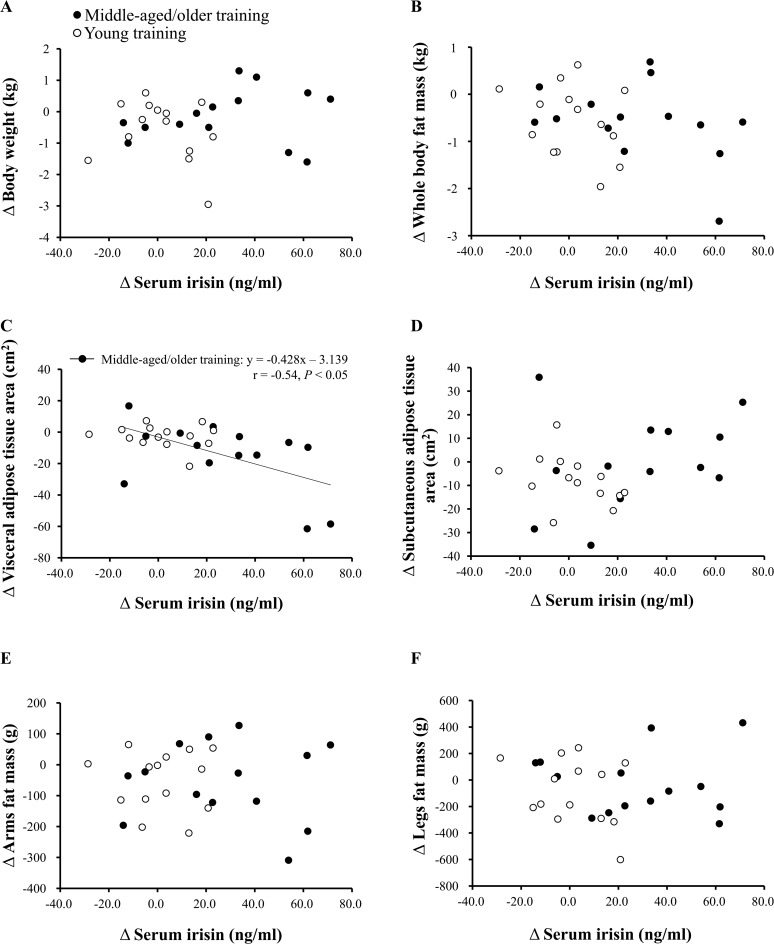
Associations between change in serum irisin concentration and change in body weight (A), whole-body fat mass (B), abdominal visceral adipose tissue (C), abdominal subcutaneous adipose tissue (D), arm fat mass (E), and leg fat mass (F). Solid circles: middle-aged/older training group, open circles: young training group.

At baseline, serum irisin concentration was not correlated with body weight, abdominal subcutaneous and visceral adipose tissue areas, whole-body fat, or arm and leg fat. In the young and middle-aged/older training groups, the change in serum irisin concentration negatively correlated with the change in abdominal visceral adipose tissue area (r = −0.57, *P* < 0.01, Fig. 3-C). Furthermore, in the middle-aged/older training group only, there was a significant correlation between the change in serum irisin concentration and the change in visceral adipose tissue area (r = −0.54, *P* < 0.05), whereas no such correlation was seen in the young training group. We observed no significant association of the change in serum irisin with change in body weight, abdominal subcutaneous adipose tissue area, whole-body fat, or arm and leg fat (Fig. 3-A, B, D, E, F).

## Discussion

In this study, we investigated the effects of endurance training on circulating irisin levels in healthy young and middle-aged/older adults. Circulating irisin levels decreased with age; however, the circulating irisin levels in middle-aged/older adults were elevated after the 8-week endurance-training intervention, and this change occurred concomitantly with improvements in body composition, e.g., reductions in whole-body fat mass and abdominal visceral adipose tissue area. The change in circulating irisin levels before and after 8-week endurance training was negatively correlated with the change in visceral adipose tissue, whereas changes in other parameters of body composition were not associated with irisin levels. Therefore, these results suggest that the exercise training-induced increase in circulating irisin is associated with the reduction in visceral fat following endurance training in healthy middle-aged/older adults.

In an animal study, moderately elevated levels of circulating irisin induced expression of *UCP1* mRNA in white adipose cells via activation of PPAR-α protein, leading to browning of white adipocytes (beige-cell formation) and thereby increasing energy expenditure through thermogenesis [5]. Therefore, a high level of circulating irisin may be associated with resistance to fat accumulation throughout the body. This study demonstrates, for the first time, that elevation of circulating irisin levels by endurance training is associated with deceasing visceral fat area in middle-aged/older adults. Higher circulating irisin levels are predicted to cause greater energy expenditure throughout the body, which may contribute to the reduction of visceral fat in middle-aged/older adults who undergo exercise training. In fact, circulating irisin levels were positively correlated with 24-h energy expenditure in some subjects, specifically those whose energy expenditure was greater than estimated by the fat free mass-based equation [6].

Our results show that the endurance training-induced change in circulating irisin levels was negatively correlated with the change in visceral fat, but not with changes in body weight, abdominal subcutaneous fat, whole-body fat, or arm and leg fat mass. In the middle-aged/older training group, abdominal subcutaneous adipose tissue area and leg fat mass did not change after the 8-week endurance-training intervention. Visceral adipose tissue is more metabolically active and sensitive to lipolysis [13, 14], and thus may contribute more to the weight reduction than subcutaneous adipose tissue. After initial weight loss due to reduction of visceral adipose tissue, further intervention may lead to an overall reduction in body fat [14]. Therefore, the reduction in visceral adipose tissue following the 8-week endurance training program was greater than the reduction in subcutaneous adipose tissue. Although the changes in visceral adipose tissue and irisin were correlated in this study, longer periods of exercise intervention may cause greater reduction of fat accumulation throughout the body [15–17], with a concomitant increase in circulating irisin levels in older subjects. Further studies are required to examine the effects of longer interventions in the older adults.

Hecksteden et al. [18] reported that circulating irisin levels were not changed by 26 weeks of aerobic training consisting of walking and running at 60% of heart-rate reserve for 45 min, 3 days/week, in healthy subjects 30–60 years of age. By contrast, the findings of this study show that in the healthy middle-aged/older adults, circulating irisin levels were elevated by endurance training consisting of cycling on a leg ergometer at 60–70% VO_2peak_, whereas the irisin levels of young adults did not change after the same exercise intervention. In the healthy middle-aged/older group of this study, 24 out of 28 subjects were aged 60 and over. Therefore, it is possible that age differences in the cohorts used in the two studies explain the discrepancies in their results. Additionally, multiple recent studies reported no change in circulating irisin level after exercise training [19–21]. In this case, the inconsistency with our results might be explained by differences in the types of exercise used in the interventions or the characteristics of the subjects.

In this study, we demonstrated that the aging-induced reduction in circulating irisin levels can be restored by endurance training. However, the source of the exercise training-induced increase in serum irisin levels remains unclear. *FNDC5* mRNA is expressed at higher levels in skeletal muscle than in other organs [9], and FNDC5 expression in skeletal muscle contributes significantly to circulating irisin levels [5, 9, 22]. Endurance training increases the levels of circulating irisin and muscle FNDC5 expression in older adults [5]. Timmons et al. [8] showed that muscle FNDC5 expression was higher in older adults with high cardiorespiratory fitness than in older low-fitness adults. FNDC5 protein expression is regulated via the PGC-1α signaling pathway [5]. In two previous animal studies, aging reduced the expression of PGC-1α in skeletal muscle [23, 24], and age-associated reduction in PGC-1α protein expression in skeletal muscle was reversed by endurance training [23, 24]. Lecker et al. [25] reported that muscle FNDC5 expression correlated with PGC-1α expression and VO_2peak_ in a group of heart-failure patients with high cardiorespiratory fitness, but these measurements were not correlated in a low-fitness group. Therefore, increased muscle FNDC5 expression, mediated by activation of PGC-1α signaling, might play a role in the endurance training-induced enhancement of irisin secretion in middle-aged/older adults. However, we did not investigate whether these inducers were activated by exercise training in the middle-aged/older subjects examined in this study. Future studies should examine the effects of regular exercise training on the activities of these inducers in skeletal muscle.

Previous studies revealed possible sex differences in circulating irisin concentration [9, 21]. However, other previous studies with large sample sizes demonstrated that the baseline level [7] and training response [18] of circulating irisin level did not differ between sexes. In this study, we observed no significant difference between sexes in serum irisin concentration at baseline, and training responses of serum irisin concentration were similar between sexes (data not shown). In addition, it is possible that differences in body composition between young and middle-aged/older adults influence the training responses of irisin observed in this study. Finally, to address the issues of small sample size and multiple comparisons, which represent limitations of this study, it will be necessary to conduct a randomized controlled trial with a larger sample size, and may clarify the association between the effects of training on serum irisin level and visceral fat is age specific. Elucidating the mechanisms of variability in exercise-induced reduction of body fat will facilitate development of more effective exercise prescriptions for metabolic syndrome and/or obesity.

In summary, we investigated the effects of an 8-week endurance training program on circulating irisin levels. After the 8-week intervention, circulating irisin levels were significantly elevated in healthy middle-aged/older adults, but were not altered in young adults. In addition, the increase in circulating irisin levels was significantly correlated with the reduction of visceral adipose tissue area resulting from endurance training. Although the detailed mechanisms should be investigated in future studies, our results suggest a possible role for secreted irisin in the exercise-induced alteration of abdominal visceral fat in middle-aged and older adults.
